# Efficacy and safety of two-stage revision for patients with culture-negative versus culture-positive periprosthetic joint infection: a single-center retrospective study

**DOI:** 10.1186/s12891-024-07259-7

**Published:** 2024-02-20

**Authors:** Hanpeng Lu, Wenqi Wang, Hong Xu, Kai Zhou, Zongke Zhou

**Affiliations:** 1 Department of Orthopaedic Surgery, West China Hospital, Sichuan University, Chengdu, 610041 China; 2 West China School of Medicine, Sichuan University, Chengdu, China

**Keywords:** Periprosthetic joint infection, Culture negative, Culture positive, Two-stage revision, Reinfection rate

## Abstract

**Background:**

The safety and efficacy of two-stage revision for culture-negative PJI remain controversial. This study analyzed outcomes after two-stage revision in patients with culture-negative and culture-positive periprosthetic joint infection (PJI) during follow-up lasting at least two years.

**Methods:**

Data were retrospectively analysed patients who underwent hip or knee revision arthroplasty from January 2008 to October 2020 at our medical center. The primary outcome was the re-revision rate, while secondary outcomes were the rates of reinfection, readmission, and mortality. Patients with culture-negative or culture-positive PJI were compared in terms of these outcomes, as well as survival time without reinfection or revision surgery, based on Kaplan‒Meier analysis.

**Results:**

The final analysis included 87 patients who were followed up for a mean of 72.3 months (range, 24–123 months). The mean age was 58.1 years in the culture-negative group (*n* = 24) and 59.1 years in the culture-positive group (*n* = 63). The two groups (culture-negative versus culture-positive) did not differ significantly in rates of re-revision (0.0% vs. 3.2%, *p* > 0.05), reinfection (4.2% vs. 3.2%, *p* > 0.05), readmission (8.4% vs. 8.0%, *p* > 0.05), or mortality (8.3% vs. 7.9%, *p* > 0.05). They were also similar in survival rates without infection-related complications or revision surgery at 100 months (91.5% in the culture-negative group vs. 87.9% in the culture-positive group; Mantel‒Cox log-rank χ^2^ = 0.251, *p* = 0.616).

**Conclusion:**

The two-stage revision proves to be a well-tolerated and effective procedure in both culture-negative and culture-positive PJI during mid to long-term follow-up.

## Introduction

Prosthetic joint infection (PJI) is one of the most serious complications after total joint arthroplasty. It prolongs hospitalization and increases medical costs as well as the risk of morbidity and mortality [1]. PJI occurs in approximately 1% of patients undergoing hip arthroplasty and 1–2% of patients undergoing knee arthroplasty [2], which translates to large patient numbers given that in the US alone, the numbers of primary total hip arthroplasties (THAs) and primary total knee arthroplasties (TKAs) will increase to 635,000 and 1260,000, respectively, by 2030 [3].

There are several criteria for diagnosing PJI, including microbial culture [4–6], and a positive culture test makes the diagnosis more certain and can guide the choice of antibiotic for treatment. Culture-negative PJI places a substantial burden on both clinicians and patients [7], as indicated by increased hospitalization, mortality and adverse drug reactions [8]. Considering the significant negative influence of culture-negative PJI, it is necessary to isolate pathogens as much as possible. However, 5–45% of patients with PJI are still culture-negative [7]. A negative culture test in these patients may reflect error, previous use of antibiotics, and biofilm formation [9]. Two-stage revision is considered as the gold standard for patients with PJI [10]. However, the effect of two-stage revision for culture-negative patients is controversial. On the one hand, some researchers reported culture-negative patients with PJI had similar outcomes to culture-positive patients [11–13]. On the other hand, some researchers reported culture-negative patients had worse outcomes and more risk of re-infection [14], while another suggested that culture-negative patients had higher success rate of infection control [15]. Therefore, more cohort researches are required from different joint center to further determine the efficacy and complications of two-stage revision for culture-negative patients.

To help address this controversy, the present study retrospectively investigated the efficacy and safety of two-stage revision in patients with culture-negative or culture-positive PJI, all of whom were followed up for at least two years.

## Materials and methods

### Study design and patients

This retrospective cohort study was approved by the Ethics Committee of West China Hospital, Sichuan University (approval no. 2022 − 1545). There was no informed consent for this study because the data we needed were anonymous and harmless to patients. We evaluated all patients at our center, which is the largest joint center in western China, who underwent revision surgery between January 2008 and October 2020 were screened for revision surgery due to PJI and diagnosed based on the 2013 criteria from the Musculoskeletal Infection Society (MSIS) [5]. We included only 544 patients for whom adequate medical data could be extracted from the hospital electronic records system during a follow-up of at least two years. We excluded 2 patients who had a history of malignant tumors or infection in other parts of the body. We also excluded 297 patients who experienced aseptic loosening. In the end, we selected patients who underwent complete two-stage revision surgery due to PJI and excluded patients who did not complete two-stage revision or who underwent other treatment strategies, such as one-stage revision, joint fusion, or the “DAIR” protocol of debridement, antibiotics, irrigation, or prosthesis retention.

Culture-negative PJI was defined as a case in which cultures of joint aspirate and surgical samples were negative according to published criteria [16]. We conducted additional fungal and mycobacterial cultures according to the patients’ conditions (medical history, physical examination, imaging tests, and hematological investigations) evaluated by senior doctors.

### Surgical and antimicrobial procedures

At our institution, two-stage revision has been established as the routine treatment for patients with PJI. Moreover, all surgeries were performed by five experienced orthopedic surgeons as described previously [17]. The first stage involved using a posterior-lateral approach to the hip joint, ensuring complete removal of the infected tissue. For patients with sinus tracts, the sinus tract was incised along its course, and the infected foci, as well as any surrounding scar tissue or granulation tissue around the joint prosthesis, were completely excised. Subsequently, hydrogen peroxide solution (3% concentration) and povidone-iodine solution (with an effective iodine concentration of 0.5%) were sequentially injected into the incision, acetabulum, and medullary cavity. The area was soaked for 10–15 min, followed by thorough irrigation with a pulse lavage gun using 6 L of normal saline to completely remove debris, infection, and devitalized tissue. This process was repeated two to three times to create a nearly sterile fresh wound surface. A homemade antibiotic-loaded bone cement, which was prepared in-house using Palacos polymethylmethacrylate cement (Biomet, Warsaw, IN, USA) containing 2 g of vancomycin and 1 g of gentamicin per pack, was then placed. Before reimplantation of the prosthesis, patients were advised to perform muscle strength exercises to prevent muscle atrophy.

All patients underwent preoperative joint aspiration under ultrasound guidance to obtain pathological evidence. During surgery, synovial fluid and at least five tissue samples from the joint were obtained for pathogen culture. After the tissue was sampled, intravenous broad-spectrum antibiotics were administered intraoperatively. All the synovial fluid was collected for cell counting and incubation in blood culture bottles.

To culture the bacteria, standard culture methods were employed. If the samples did not yield positive results using standard culture, an enrichment culture method was utilized. The standard culture method was conducted at 35 °C/5% CO_2_ for 24 h. The enrichment culture was carried out at 35 °C in ambient air for 5 days. If no growth occurred during the enrichment culture, the sample was considered culture negative. If tissue samples were culture positive, drug sensitivity tests were performed, and based on the results, specific antibiotics were administered. If culture-negative results were obtained, patients were administered vancomycin and cephalosporins. All patients usually received intravenous antibiotics for six weeks, followed by oral treatment for 2–4 weeks depending on their condition [18–20] after the first-stage surgery.

The timing of the second stage of revision surgery, reimplantation, was decided based on comprehensive analysis of each patient’s condition (no fever, absence of local inflammation, including swelling and local tenderness around the joint) [21] and relevant serum indicators, such as C-reactive protein levels and erythrocyte sedimentation rate. After three consecutive normal results of the aforementioned criteria were obtained, a second-stage revision surgery was typically performed. The same surgical approach was used to access and remove the cement spacer. Like in the first stage, thorough debridement was performed. Hydrogen peroxide solution (3% concentration), povidone-iodine solution (with an effective iodine concentration of 0.5%), and pulsatile irrigation with normal saline were used. Appropriate hip revision prostheses were then implanted after irrigation. If there was any bone defect, bone graft reconstruction was performed prior to implantation of the revision prosthesis. Antibiotics were delivered intravenously during the second stage, just as during the first stage, until microbial outcomes were obtained from intraoperative samples. If tissue samples from the second stage were culture-positive, specific intravenous antibiotics were administered within four weeks, followed by oral treatment for another four weeks. If culture-negative results were obtained, patients were given intravenous antibiotics for one week, followed by oral treatment for two weeks.

### Outcomes and follow-up

Patient sex, age, height, weight, body mass index and culture-negative or culture-positive diagnosis data were extracted from hospital records. Preoperative laboratory test data were also extracted. Patients were followed up at 3 weeks, 2 months, 6 months and 12 months after surgery and once a year thereafter. During follow-up, the patients were assessed for the primary outcome of revision surgery, as well as for the secondary outcomes of reinfection, readmission related to PJI, complications, and mortality. Patients were defined as free from infection if they satisfied the following criteria by the last follow-up [22]: (1) had no recurrence of infection caused by the same bacteria, with satisfactory wound healing without fistula, drainage or pain; (2) had no repeat surgery for infection caused by the same bacteria; and (3) had no mortality that could be related to PJI, such as mortality due to sepsis or necrotizing fasciitis.

### Statistical analysis

The data were analyzed using SPSS 28.0 (IBM, Chicago, IL, USA). The data were reported as the means and standard deviations or as proportions. Differences between patients with culture-negative or culture-positive PJI were assessed for significance using the independent-samples *t* test for continuous data showing a normal distribution, the Mann‒Whitney U test for continuous data showing skew, or the chi-squared test or Fisher exact test for categorical data.

Survival curves for patients with infection-related complications or who underwent revision surgery were generated using the Kaplan‒Meier method, and differences between the curves for patients with positive-negative or -positive PJI were assessed for significance using the Mantel‒Cox log-rank test. The endpoints for such curves were defined as either (1) readmission for infection-related complications or (2) prosthesis revision because of infection, loosening based on radiography, or any other implant issues.

Differences were considered significant if two-sided *p* < 0.05.

## Results

**Fig. 1:**
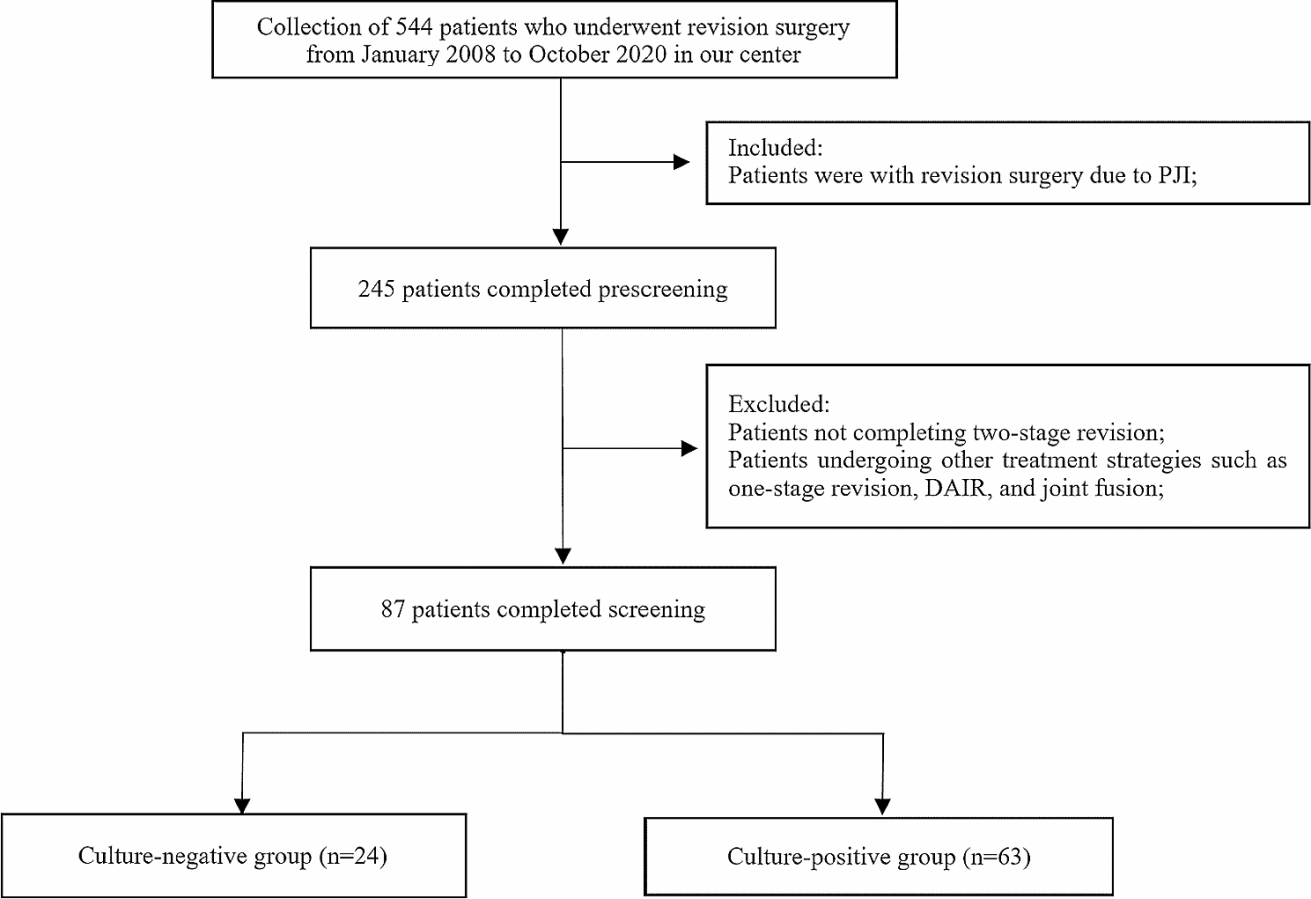
Flow diagram of the study

Among the 544 patients who underwent knee or hip revision surgery at our joint center between January 2008 and October 2020, a total of 245 patients were selected for inclusion in our study due to revision surgery for periprosthetic joint infection (PJI). Based on our inclusion criteria, we excluded patients who underwent one-stage revision (35 patients); joint fusion (2 patients); or the “DAIR” protocol involving debridement, antibiotics, irrigation, and prosthesis retention (32 patients); and who did not undergo reimplantation (89 patients). Ultimately, a total of 87 patients were enrolled in our study, 24 of whom were diagnosed with culture-negative PJI and 63 with culture-positive PJI (Fig. 1). A sinus communicated with the joint in 32 patients. All patients were followed up for an average of 72.3 months (range 24–123 months, SD 30.4). There was no significant difference in the time to reimplantation between the two groups (*p* = 0.609).

The patients with culture-negative or culture-positive PJI did not significantly differ in terms of mean age; distribution of PJI in knees or joints; smoking status; alcohol intake; or incidence of hypertension, diabetes, cardiovascular disease, chronic obstructive pulmonary disease, or inflammatory disease (Table 1). Among the 24 patients with culture-negative PJI, microbiology cultures yielded negative results; however, some histology results indicated the presence of inflammation. Twenty-two patients were positive for inflammation according to histology, 17 were positive for periprosthetic purulence during surgery, and 6 were positive according to sinus examination (Table 2). Among the 63 patients with culture-positive PJI, 54 were infected with gram-positive bacilli, three with gram-negative bacilli, two with mycobacteria, two with multiple microbes, one with anaerobic bacteria, and one with fungus (Table 3). The two most common pathogens were *Staphylococcus epidermidis* (*n* = 30), followed by *S. aureus* (*n* = 16).

**Table 1:** Clinicodemographic characteristics of patients with periprosthetic joint infection, stratified by culture test results

Characteristic	Total (*n* = 87)	Culture-negative (*n* = 24)	Culture-positive (*n* = 63)	*p* value
Female, n (%)	44 (50.6)	16 (66.7)	28 (44.4)	0.064
Age, years	58.8 ± 13.8	58.1 ± 12.9	59.1 ± 14.2	0.765
Body mass index, kg/m^2^	23.8 ± 3.9	22.4 ± 3.6	24.2 ± 3.9	0.087
Sinus	32 (36.8)	6 (25.0)	16 (41.3)	0.160
Follow-up	72.3 ± 30.4	78.5 ± 29.6	70.0 ± 30.6	0.243
Time to reimplantation*	7 (7)	7 (7)	7 (7.25)	0.609
Comorbidities				
Smoking	25 (28.7)	4 (16.7)	21 (33.3)	0.125
Alcohol	22 (25.3)	4 (16.7)	18 (28.6)	0.254
Hypertension	27 (31.0)	7 (29.2)	20 (31.7)	0.816
Diabetes	12 (13.8)	2 (8.3)	10 (15.9)	0.362
Cardiovascular disease	4 (4.6)	0 (0.0)	4 (6.3)	0.489
Chronic obstructive pulmonary disease	3 (3.4)	0 (0.0)	3 (4.8)	0.558
Inflammatory disease	8 (9.2)	5 (20.8)	3 (4.8)	0.057
Joint involved				0.169
Hip	57 (65.5)	13 (54.2)	44 (69.8)	-
Knee	30 (34.5)	11 (45.8)	19 (30.2)	-

The median (interquartile range) was used to describe *; apart from that, values are described with n (%) or mean ± SD

**Table 2:** Positive diagnostic criteria identified in the culture-negative group

Criteria	Patients (n)
Sinus, n (%)	6 (25.0)
Histopathology (showing inflammation), n (%)	22 (91.7)
Periprosthetic purulence observed at operation, n (%)	17 (70.8)

**Table 3:** Isolated microorganisms in the culture-positive group

Microorganisms classification	Patients, n (%)	Microorganisms	Specific quantity, n
Gram-positive	54 (85.7)	Staphylococcus epidermidis	29
		Staphylococcus aureus	16
		Other Staphylococcus	5
		Streptococcus	1
		Enterococcus	3
Gram-negative	3 (4.8)	Escherichia coli	1
		Enterobacter cloacae	2
Anaerobes	1 (1.6)	Staphylococcus saccharolyticus	1
Mycobacterium	2 (3.2)	Mycobacterium tuberculosis	2
Polymicrobial	2 (3.2)	Pseudomonas aeruginosa/Staphylococcus aureus	1
		Staphylococcus epidermidis/ Mycobacterium tuberculosis	1
Fungus	1 (1.6)	Candida albicans	1

Other staphylococci include Staphylococcus hemolytic (1 case), Staphylococcus hominis (2 cases), Staphylococcus warneri (1 case), and Staphylococcus caprae (1 case)

During the follow-up period, two patients with culture-positive PJI underwent re-revision surgery, while no patients with culture-negative PJI required re-revision surgery. In addition, three patients, one with culture-negative PJI and two with culture-positive PJI, experienced reinfection after two-stage revision surgery (Table 4). Two patients were treated with the DAIR protocol, while one patient with culture-positive PJI underwent re-revision surgery.

**Table 4:** Outcomes of patients with periprosthetic joint infection during follow-up

Outcome	Total (*n* = 87)	Culture-negative (*n* = 24)	Culture-positive (*n* = 63)	*p* value
Re-revision	2 (2.3)	0 (0.0)	2 (3.2)	> 0.05
Aseptic loosening	1 (1.1)	0 (0.0)	1 (1.6)	> 0.05
Dislocation of hip	1 (1.1)	0 (0.0)	1 (1.6)	> 0.05
Reinfection	3 (3.4)	1 (4.2)	2 (3.2)	> 0.05
Repeat first-stage surgery	1 (1.1)	0 (0.0)	1 (1.6)	> 0.05
Readmission	7 (8.0)	2 (8.4)	5 (8.0)	> 0.05
Within 90 days	3 (3.4)	1 (4.2)	2 (3.2)	> 0.05
After 90 days	4 (4.6)	1 (4.2)	3 (4.8)	> 0.05
Mortality	7 (8.0)	2 (8.3)	5 (7.9)	> 0.05

Values are n (%), unless otherwise noted

**Fig. 2:**
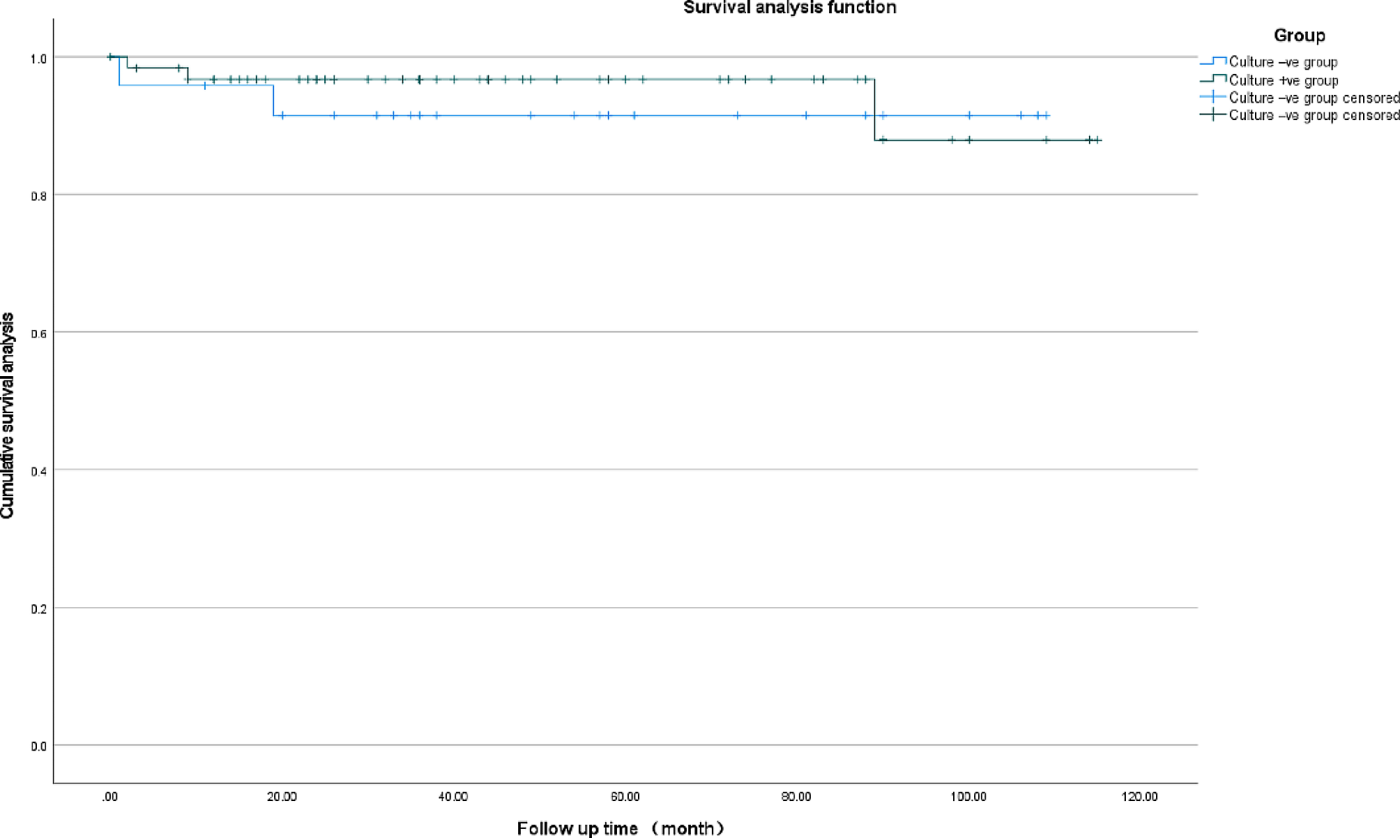
Kaplan‒Meier curves of survival free from readmission due to infection or reoperation showing similar survival rates between the culture-negative and culture-positive groups at 100 months

One patient with culture-positive PJI experienced aseptic loosening and underwent re-revision surgery. One patient with culture-positive PJI suffered dislocation after hip revision surgery, while another underwent repeat first-stage surgery due to fungal infection. Two patients with culture-negative PJI (8.4%) were readmitted to our center for complications, compared to five patients with culture-positive PJI (8.0%) were readmitted. The percentages of patients free from infection-related complications at 100 months were 91.5% for patients with culture-negative PJI and 87.9% for patients with culture-positive PJI (Fig. 2); these percentages did not significantly differ based on the Mantel‒Cox log-rank test (χ^2^ = 0.251, *p* = 0.616).

No patients in the study died within one year after surgery. Two patients with culture-negative PJI and five with culture-positive PJI died during follow-up, but neither of the deaths was attributed to the surgery.

## Discussion

This retrospective study of a relatively small sample suggests that the efficacy and safety of two-stage revision surgery are similar for patients with culture-negative or culture-positive PJI. This has been an open question in the literature, given that negative culture tests may indicate an infection that is resolving due to previous antibiotic use [16, 23, 24], formation of biofilms that can resist antibiotic treatment [25], or incorrect sampling or sample handling [26, 27], all of which may affect outcomes after two-stage revision surgery.

Our cohort showed approximately 90% survival from reinfection and re-revision by 100 months after surgery, consistent with survival rates of 80–90% for patient cohorts in the U.S [28–31]. . Furthermore, we found that this high survival was similar between patients with culture-negative or culture-positive PJI, consistent with the findings of other studies [11–13]. One study associated culture-negative PJI with worse outcomes and a greater rate of salvage procedures than culture-positive PJI [14]; however, these findings may be attributed to the fact that some patients in that study underwent treatments other than two-stage revision, which may be less effective against culture-negative PJI [15, 32]. These considerations imply that two-stage revision may be the most effective treatment for culture-negative PJI.

Vancomycin and cephalosporins are the antibiotics most commonly used to treat patients with culture-negative PJI who are undergoing two-stage revision [33]. We achieved an infection eradication rate of 91.5% with these antibiotics in the present study, consistent with the eradication rates of 70–92% reported for patient cohorts [15, 16, 34, 35]. The pathogenesis of culture-negative PJI is thought to be due to fungal and mycobacterial infections in more than 85% of all cases [36]; it is worth noting that the standard treatment courses of these conventional antibiotics are often ineffective against mycobacteria and fungi. Therefore, if infection with fungi or mycobacteria is suspected, samples should be cultured on conditioned media or subjected to next-generation sequencing to ensure timely diagnosis and treatment [37, 38].

Our findings should be interpreted with caution given the relatively small sample size from a single center. Our retrospective study meant that we could not analyze certain clinically relevant questions, such as whether previous use of antibiotics increases the risk of culture-negative PJI [39, 40]. In addition, to explore the efficacy of complete two-stage revision in the treatment of culture-negative PJI, patients were strictly screened, which resulted in a relatively small number of patients ultimately being selected, limiting the generalizability of the conclusions. However, due to the limited number of relevant studies available and considering that our hospital serves a diverse range of patients as a medical center in Southwest China, our results are still suggestive. Among the excluded patients, many had planned for a two-stage revision but did not undergo prosthesis reimplantation due to factors such as limited access to healthcare resources, lack of family support, or severity of the disease. This may introduce bias between the two groups, highlighting the need for further prospective research to investigate the differences in treatment outcomes between culture-positive and culture-negative PJI patients. In addition, while we followed up patients for at least two years, further work should examine the efficacy and safety of two-stage revision surgery over longer periods for patients with culture-negative PJI. Finally, implant sonication is an effective method for reducing culture negativity. However, a significant limitation of our study is that explanted implants were not subjected to sonication.

Despite concerns regarding culture-negative PJI, our study showed that with the implementation of appropriate management strategies, two-stage revision may have comparable efficacy and safety in both culture-negative and culture-positive patients with PJI.

## Data Availability

The original data presented in the study are included in the article, and no additional data remain under study.
